# The effectiveness of medical nutrition therapy for people at moderate to high risk of cardiovascular disease in an Australian rural primary care setting: 12-month results from a pragmatic cluster randomised controlled trial

**DOI:** 10.1186/s12913-025-13096-8

**Published:** 2025-07-16

**Authors:** Tracy L. Schumacher, Anna Jansson, Jaimee Herbert, Erin D. Clarke, Carissa Alderton, Penny Milson, Christopher Oldmeadow, Leanne J. Brown, Megan E. Rollo, Annabelle Williams, M. Com Nutr, Michelle Guppy, Andrew Boyle, Shanthi Ramanathan, Jennifer May, John Attia, Clare E. Collins

**Affiliations:** 1 Department of Rural Health, College of Health Medicine and Wellbeing, University of Newcastle, Tamworth, NSW 2340 Australia; 2 Food and Nutrition Research Program, Hunter Medical Research Institute, New Lambton Heights, NSW 2305 Australia; 3 School of Health Sciences, College of Health Medicine and Wellbeing, University of Newcastle, Callaghan, NSW 2308 Australia; 4 Data Sciences, Hunter Medical Research Institute, New Lambton Heights, NSW 2305 Australia; 5 School of Population Health, Faculty of Health Sciences, Curtin University, Bentley, WA 6102 Australia; 6 Hunter New England Central Coast Primary Health Network, Tamworth, 2340 Australia; 7 School of Rural Medicine, University of New England, Armidale, NSW 2350 Australia; 8 School of Medicine and Public Health, College of Health Medicine and Wellbeing, University of Newcastle, Callaghan, NSW 2308 Australia; 9 Heart and Stroke Research Program, Hunter Medical Research Institute, New Lambton Heights, NSW 2305 Australia; 10 Department of Cardiovascular Medicine, John Hunter Hospital, Locked bag 1, Newcastle, NSW 2310 Australia; 11 Research Impact Platform, Hunter Medical Research Institute, New Lambton Heights, NSW 2305 Australia

**Keywords:** Cardiovascular disease, Rural health, Primary health care, Medical nutrition therapy, Telehealth, Dietitian

## Abstract

**Purpose:**

To reduce risk of cardiovascular disease (CVD) in adults, as assessed by primary care doctors in rural NSW, Australia. Medical nutrition therapy (MNT) was delivered by Accredited Practicing Dietitians (APDs) using telehealth.

**Methods:**

The study was a 12-month pragmatic cluster randomised controlled trial. All primary care practices (PCPs) within a large rural region were invited to participate, with enrolled practices stratified based on rurality and practice size. Patients at moderate to high CVD risk were recruited via practices. Usual care (UC) was provided by the patient’s general practitioner (GP). In addition to UC, the intervention group received two hours of MNT telehealth (video calls) consultations from an APD during five sessions over 6 months. The primary outcome was total serum cholesterol. Secondary outcomes included LDL cholesterol, triglycerides, blood glucose control, blood pressure, weight and waist circumference. Changes were analysed using Bayesian linear mixed models and posterior probability.

**Findings:**

Sixteen PCPs recruited 132 eligible participants (*n* = 91 intervention, *n* = 41 UC), with 79% (72/91) and 80% (33/41) respectively completing a primary or secondary outcome. No significant differences were found between groups for total cholesterol, LDL cholesterol or blood pressure at 12-months. However, the intervention group had significant improvements in blood glucose control (HbA1c: -0.16%, 95%CI: -0.32, -0.01) and decreased body weight (-2.46 kg, 95%CI: -4.54, -0.41) compared to UC at 12-months.

**Conclusions:**

Results indicate that two hours of MNT delivered by an APD via telehealth is a synergistic adjunct therapy to support the usual care provided by GP, with benefits continuing to 12-months.

**Supplementary Information:**

The online version contains supplementary material available at 10.1186/s12913-025-13096-8.

## Introduction

There are a multitude of behavioural (i.e., smoking, diet, physical activity, and alcohol) and biomedical (i.e., hypertension, abnormal blood lipids, diabetes and overweight/obesity) risk factors that can increase the likelihood of developing cardiovascular disease (CVD), with many being modifiable [1, 2]. Poor diet quality, comprising of diets that are high in processed meats, sodium and sugar, and low in fruits, vegetables and wholegrains, is one of the leading risk factors for CVD globally [3]. Australian adults have poor diet quality with a third of their diet coming from unhealthy foods and 94% not meeting fruit and vegetable recommendations [2, 4]. People living rurally experience on average, more risk factors including higher total energy intakes, compared to those living in metropolitan areas [5]. Poor diet quality also contributes to risk of type 2 diabetes mellitus (T2DM), a major chronic condition in Australia that is also more prevalent in rural and remote areas [6] and that results in a two-fold increase in CVD risk [7, 8]. If modifiable risk factors for CVD were equal between rural and metropolitan populations, CVD related deaths would be reduced by 38% [5].

Lifestyle modifications and pharmacotherapy are two key interventions for managing elevated CVD risk factors [9, 10]. CVD risk is generally determined using several factors e.g. age, sex, smoking status, blood pressure, cholesterol levels and diabetes status [9–12]. Regardless of the level of risk, guidelines from several developed countries state that individuals should be encouraged, supported and advised on healthy lifestyle modifications, and pharmacotherapy should be considered depending on clinical context in those at higher risk of developing CVD [13]. Lifestyle modifications are often personalised to improve risk factors specific to the individual; however, small, positive changes in diet and exercise can reduce risks for a wide range of health conditions while also improving CVD risk [14–17].

Access to healthcare is a strong social determinant of health including its influence on developing CVD and becomes particularly relevant in rural Australia where accessing primary health care has been linked to ongoing issues of socioeconomic disadvantage, and an insufficient and unevenly distributed health workforce [18–21]. People living in rural Australia are more likely to experience socioeconomic disadvantage, which adds to the burden of disease with the most socioeconomically disadvantaged being 1.2 times more likely to have high blood pressure and 1.7 times more likely to have CVD [21, 22]. General practitioners (GPs; equivalent to primary care physicians in US) are often the primary healthcare professional responsible for managing CVD in Australia, especially identifying and managing risk factors and early signs of illness [1]. GPs will often oversee medication management, provide lifestyle advice and, if applicable, provide referrals to other healthcare professionals including dietitians. While dietitians can provide guidance of how to manage diet and nutrition for people affected by health conditions including CVD, they are often underutilised in chronic disease management [23]. One study from the United States reported that primary care physicians are generally interested in integrating dietitians into care, but barriers such as not having a dietitian on site, difficulties of finding dietitians in rural areas, feeling uncertain how to find a local practitioner and additional costs for the patient prohibits referrals [24].

While there are estimates of the spread of the GP workforce across Australia, data for the Accredited Practising Dietitian (APD) [25] workforce is poor, especially for rural areas. The APD workforce in Australia has been estimated to be 24 APDs per 100,000 people, with 78.6% of the workforce residing in a metropolitan area [26]. This is compared to 118.6 GPs per 100,000 individuals in major cities, and between 129.5 and 80.3 in large to small rural towns in New South Wales, the state where this study was conducted [27]. To increase access to APDs in rural areas, telehealth was seen as a promising platform for delivering dietary interventions. Telehealth systems, including video calls, support the delivery of healthcare between practitioners and recipients in separate locations [28, 29]. However, no ‘real-world’ trials had been conducted with the aim of enhancing the accessibility and provision of dietetic services for CVD care in rural Australia, and no dietitian-led telehealth interventions targeting CVD care in rural areas had been evaluated using an RCT design [30].

Medical Nutrition Therapy (MNT) delivered by an APD, is an effective individualised evidence-based intervention that targets diet-related behaviours [31, 32]. Lifestyle management, including MNT is recommended as a first line treatment according to the Royal Australian College of General Practitioners endorsed guidelines for the management of absolute CVD risk [33, 34]. Telehealth is a promising intervention delivery platform for rural areas, given the ability to provide services across vast geographical distances. Despite this, only a handful of studies have used telehealth in rural Australia for the delivery of nutrition interventions and of these, none had implemented a personalised MNT intervention [35]. Further, a recent systematic review on personalised nutrition interventions in people at elevated risk of CVD found that no studies were conducted in Australia [36], highlighting the need for more research on the application of telehealth MNT delivered by APDs in rural Australia. Therefore, the primary aim of this randomised controlled trial (RCT) was to reduce total cholesterol in patients living in rural locations assessed as having high to medium CVD risk using an MNT intervention delivered by an APD via telehealth. The secondary aim was to reduce other modifiable risk factors related to CVD health, including LDL cholesterol, triglyceride, blood pressure, blood glucose control, weight and waist circumference.

## Methods

### Study design

Healthy Rural Hearts (HealthyRHearts) was a pragmatic 12-months parallel group cluster RCT and reported according to the 2010 CONSORT statement: extension to cluster randomised trials [37], trials affected by COVID-19 [38], and the TIDieR checklist for better reporting of interventions [39]. Detailed overall and MNT study protocols have been published elsewhere [40, 41]. This project received ethics approval from the University of Newcastle Human Research Ethics Committee (H-2021-0193), safety approvals from the University of Newcastle Health and Safety Committee (49/2021) and was prospectively registered with the Australian New Zealand Clinical Trials Registry (ACTRN:12621001495819||http://www.anzctr.org.au/, 03/11/2021).

### Study setting

This study was conducted in rural primary care practices located within the area covered by the Hunter New England Central Coast Primary Health Network (HNECC PHN) [42]. The level of remoteness of geographical areas were defined using the Modified Monash Model (MMM) [43], a framework with classifications including metropolitan (MM1), regional (MM2), rural (MM3-5), remote and very remote areas (MM 6–7). Areas included within this study region were categorised as MM3 to MM6, with town population sizes up to approximately 50,000 inhabitants. Statistics from 2021 for the entire HNECC PHN region indicate that approximately 82% of the population were born in Australia, with 5% having a non-English speaking background, compared to 18% for the rest of Australia. Approximately 6% of the population identified as Aboriginal or Torres Strait Islander, compared to 3% for the rest of New South Wales [44].

### Recruitment and registration of primary care practices and general practitioners

All primary care practices and/or GPs that (i) were based in an area classified as MM3-MM6 within the region covered by the HNECC PHN, (ii) operated at minimum one day per week, and (iii) had an active de-identified data sharing agreement with the PHN, were invited to participate. At the time of recruitment planning (9th Feb 2021), 76.8% of the practices in MM3-6 areas covered by the HNECC PHN held this agreement and were considered eligible for recruitment.

Rural-based recruitment officers attempted to contact all eligible practices. The practice manager, or person acting in the capacity of practice manager was approached and offered a study information pack, which included contact details, a study fact sheet, an abbreviated study protocol, an information statement and consent forms, and other general information. Practices were given the option of a site visit, teleconference meeting, or phone conversation with the recruitment officers to discuss study details. A person responsible for the primary care practice was to provide practice consent, and at least one GP needed to register with the study. As part of the agreement, consenting practices and GPs were required to continue providing usual care to their participants throughout the duration. Practices were also asked to provide relevant information about the consenting participants, such as health conditions, medications, and anthropometric data they used when assessing heart health. Each primary care practice received a $100 payment for every participant from their practice who was assessed as eligible and randomised into a study group.

### Randomisation and blinding

Primary care practices were randomised to either intervention or usual care groups after the first study invitations were mailed to selected patients. GPs belonging to a single practice were clustered in their randomisation, due to the possibility of patients seeing another GP in the service if their own was unavailable, and to reduce any confusion regarding which intervention their patients may receive. Primary care practices were stratified based on MMM category 3–5 only (as no practices in the specified area were categorised as MM 6) and practice size (i.e., small [1–5 GPs] or medium-large [≥ 5 GPs]). A statistician external to the HealthyRHearts study developed the block randomisation procedure and uploaded it directly to the REDCap database [45, 46], which was used for data collection and management. Primary care practices and GPs were informed of their allocation at the same time of randomisation, so that they could provide care according to their clinical judgment and knowing which services were provided by the study. Participants were also informed of their study group, so they too could act based on the services they expected to receive.

### Participant recruitment and eligibility

Eligible participants were those living within an area classified as MM3 or above within the HNECC PHN region and classified by their GP as being of moderate to high risk (≥ 10%) of experiencing a CVD event over the next five years using either the 2012 CVD risk calculator [47] (based on the Framingham Risk Equation) or clinical judgement using the Guidelines for the management of Absolute cardiovascular disease risk (2012) [48]. In brief, people were deemed as eligible to participate if they had no known coronary artery disease or were deemed by their GP to be currently stable with a coronary artery disease diagnosis or had experienced no clinical events for ≥ 6 months. The full inclusion/exclusion criteria are detailed in the published protocol [40].

Participants were invited to the study by their GP, who screened their current patient list for people potentially eligible for the study and posted them a standard invitation letter with customised patient details, a study pamphlet, information statement and consent form. GPs from some practices also had the opportunity to recruit participants when they presented for appointments by providing them with a note to be exchanged for a generic letter of invitation at reception after the consultation had finished. Study flyers were also provided for display in the reception area of consenting practices, for interested patients to ask their GPs about. Participant consent included permission for relevant medical information to be shared between the study research team and GP.

After participant consent had been received, potential participants were screened for initial eligibility by study recruiting officers, who noted current medications and booked an appointment with their GP to assess their risk of heart disease. Once the heart health assessment had been completed, the GP forwarded the relevant results to the researchers. People who were assessed as moderate or high risk were enrolled in the study. When heart health assessment results included factors requiring clinical judgement, the case was referred to an advisory committee comprised of three rural GPs to determine eligibility.

### Usual care (UC) group

All participants remained under the care of their GP for the entirety of the intervention. No restrictions were placed on GPs as to what care they provided, for either pre-existing or new conditions. In addition to the initial baseline heart health assessment, participants were also asked to have an annual visit with their GP to assess factors relating to risk of CVD events on study completion. Participants in the usual care group were also invited to complete the Australian Eating Survey Heart version (AES-Heart) at baseline, 3-, 6- and 12-months. The AES-Heart is a validated online food frequency questionnaire with 194 questions relating to demographics, food preferences and food items [49, 50], that provides an automated nutrition report with personalised feedback based on responses provided. An example of the feedback report received can be found in Supplementary material one.

### Intervention group

Participants allocated to the intervention group also received usual care by their GP and the personalised AES-Heart nutrition reports. In addition, each individual was offered five MNT telehealth consultations, delivered by a rural-based APD, or APD who had worked in a rural area in the last 12 months. These sessions were not mandated. Participants were asked to complete a personalised nutrition questionnaire ahead of the initial consult to facilitate behaviour change counselling [51]. Over a period of six months, consultations were held via healthdirect, a Commonwealth funded video call service and software platform powered by Coviu [52], or over the telephone when healthdirect was not possible for the participant. Detailed information describing the development of the MNT intervention has been published elsewhere [41]. Briefly, the MNT protocols were based on the nutrition care process of assessment, diagnosis, intervention, monitoring and evaluation [53]. APDs received training from the same coordinator and were provided with the same process manual for consultations. Training included how to conduct consultations effectively via telehealth, including facilitating client behaviour change online and troubleshooting technology issues. During consultations, participants were provided with their pathology results for serum cholesterol and glycaemic control and had the chance to discuss their AES-Heart nutrition report. APDs used a range of existing behaviour change nutrition techniques based on Michie’s behaviour change wheel [54] and resources, adapted to the regional and rural context for CVD prevention [41, 51]. Additionally, communication techniques were informed by Healthy Conversation Skills principles, which aim to empower clients to identify their own strategies and solutions [55]. Topics covered within the consultations were guided by the participant’s concerns and counselled for by the APD in the context of optimising cholesterol levels and overall heart disease risk. A broad range of resources were identified as possible topics of interest and ranged from how nutrition can affect cholesterol, triglycerides and glycaemic control (diet/disease relationship); lowering serum cholesterol through nutrition, such as soluble fibres, plant sterols and long chain fatty acids; healthy eating and nutritional adequacy; sources of nutrients, including sodium, through to portion sizes; identifying habits and; meal planning.

After each consultation, the APD provided a summary of the session, including action plans and resources via email to the participant. A sample of consultations were recorded to assess APD intervention fidelity, with fidelity analysis yet to be performed. A consultation schedule, including key content, is included in Supplementary material two. Consultations were scheduled at baseline (30 min), + 2 weeks (20 min), + 4 weeks (20 min), 3 months (20 min) and at 6 months (30 min).

### Assessments and outcomes

A schedule of primary and secondary outcome data collected at the study time points of baseline, 3-, 6- and 12-months, and their assessment details can be found in Supplementary material two.

### Effects of COVID-19

This study was affected by the COVID-19 pandemic, given study recruitment involved primary care practices. Restrictions on travel and gatherings in NSW, Australia commenced in April 2020, and the last official COVID-19 lockdown occurring in the recruitment region was in August/September 2021. COVID vaccinations in Australia became available through practices from March 2021. Major disruptions and changes to workload and practice were experienced [56], adversely impacting recruitment to the study. Recruiting, training and retention of APDs to act as study dietitians were additionally affected by the second official COVID-19 lockdown in the region.

### Statistical analysis

Original sample size calculations were based on detecting a mean change in total cholesterol (primary outcome) of 0.51 mmol/L (SD = 1.1) between baseline to 12-months, with 80% power and type I error of 0.05, resulting in 74 people per group (37 men, 37 women). A dropout rate of 25% was estimated (100 per group). To account for clustering by Primary Care Practice, 10 practices per arm was assumed, with each contributing 10 participants. The design effect, assuming an ICC of 0.05, was 1.45, meaning a final sample size of 145–150 per arm, or 15 practices per arm, each contributing 10 people, was required (total of 300 participants).

The major effect of the pandemic on this study primarily resulted in a reduced sample size, inconsistent patient numbers from practices, despite a 15-month extension to the estimated 24-month trial, to recruit participants and collect follow-up data. To account for this, linear mixed models were changed to using a Bayesian approach [57].

Demographic and descriptive statistics were performed using Stata/IC v16.1. The mean change from baseline in the primary and secondary outcomes were compared between groups using Bayesian linear mixed models in R (v4.3.2). Models included fixed categorical effects for time, group, group by time interaction, age, and sex. Other variables adjusted for in outcome measures were predetermined from the literature. The model included random intercepts for individual participants to model repeated measures over time, and the primary care practice from which they were recruited, to account for the cluster randomised design. Estimates from the joint posterior distribution were obtained through MCMC sampling (the No-=UTurn-sampler, implemented in the brms package with four chains of 10000 iterations and a warmup of 1000). Flat priors were used for all fixed effects, a student t (location = 0.00, scale = 2.50) for the error standard deviation. Priors over the random effect standard deviation for individuals and GPs were set as cauchy (location = 0, scale = 5) and cauchy (location = 0, scale = 2) respectively. Convergence and stability of the Bayesian sampling has been assessed using R-hat, which should be below 1.01 [58] and Effective Sample Size (ESS), which should be greater than 1000 [59]. Model fit was assessed by taking 10 random draws from the posterior predictive distribution compared against the observed outcome distribution. Mean posterior estimates are presented, together with 95% highest density credible intervals, the probability the parameter is greater than zero for positive estimates, or less than zero for negative estimates (the probability of direction (pd)) and the evidence ratio (pd/1-pd).

## Results

### Recruitment of primary care practices, gps and participants

**Fig. 1:**
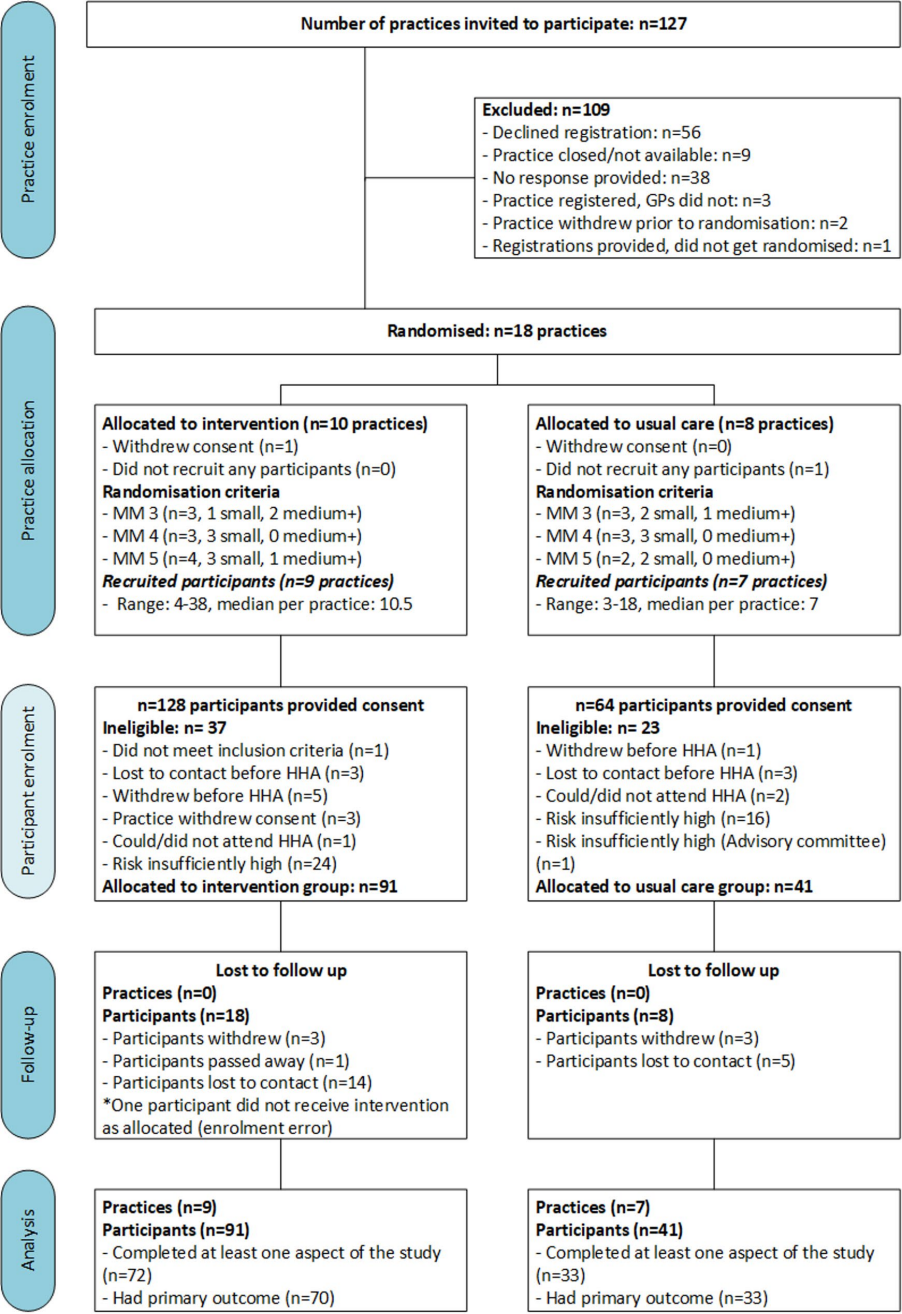
Primary care practice and participant flow chart

Recruitment of primary care practices and GPs eventually took place between February 2022 and March 2023. All eligible practices were invited to participate (*n* = 127) with 18 primary care practices being randomised to the study (14.2%) (see Fig. 1). Ten practices were randomised to the intervention group, with one withdrawing consent after inviting participants but prior to assessment of risk, citing time constraints. Eight practices were randomised to the usual care group, although one was not able to recruit participants. Participant recruitment continued from consenting practices at practice discretion, until 14 months before the study ended, except for one intervention practice that reached enrolment capacity.

A total of 192 patient consents were obtained from an extended recruitment period, which was extended for an additional four months beyond original intended date (until July 2023). Consents received per practice varied from three to 38, with a median (interquartile range) of 10 (6–15). From this, 173 people had their CVD risk assessed by their GP, with 132 determined as moderate to high risk, which included 13 determined as having sufficient risk by the advisory committee. Ninety-one and 41 participants were allocated to the intervention and usual care groups, respectively. One person did not receive the allocated intervention, due to an enrolment error. Sixty-three of the 91 enrolled participants (69%) attended all five consultations, with another 10 participants (11%) attending four consultations. Similar proportions of participants (20%) withdrew or were lost to contact in each group, and groups had comparable completion rates at 12 months (intervention: *n* = 72, 79% and control: *n* = 33, 80%).

### Baseline characteristics

Participant characteristics relating to demographics can be seen in Table 1. The median age of participants was 64.1 (interquartile range: 59.0 to 67.2), with females representing 33% of the sample (*n* = 44). Approximately half of the sample reported having private health insurance (54%). Almost half the sample reported living in town (49%), with 41% living outside of town. Most people lived with a partner (63%).

High blood pressure was reported by *n* = 86 (65%) of participants, with *n* = 91 (69%) reporting taking a medication for blood pressure control. Approximately 60% (*n* = 78) of participants reported having high cholesterol, with a similar number (*n* = 79) taking medication to control it. Approximately one in five people (*n* = 29, 22%) reported having diabetes, with *n* = 31 (24%) people taking medication to control blood glucose concentrations.

Mean fasting total cholesterol at baseline for the total sample was 4.97mmol/L (SD = 1.13) (see Table 2). All other lipid, blood sugar and blood pressure measures were above normal laboratory reference ranges and guideline recommendations [60, 61]. Mean body mass index at baseline was 31.9 (SD = 6.6), with mean weight of 94.4 kg (SD = 21.7) and mean waist circumference of 109.1 cm (SD = 14.9).

**Table 1:** Baseline HealthyRHearts study participant demographics

	Intervention (*n* = 91)		Usual Care (*n* = 41)		Total (*n* = 132)	
Level of risk at eligibility assessment						
- High (> 15% risk^a^)	53	(58.2%)	29	(70.7%)	82	(62.1%)
- Moderate (10–15% risk^a^)	30	(33.0%)	8	(19.5%)	38	(28.8%)
- Via risk assessment of advisory committee	8	(9.8%)	4	(9.8%)	12	(9.1%)
Age (years: median, IQR)	64.5	(60.7–68.7)	61.5	(57.3–65.6)	64.1	(59.0-67.2)
Sex (female: n, %)	27	(29.7%)	17	(41.5%)	44	(33.3%)
Smoking status						
- No	81	(89.0%)	33	(80.5%)	114	(86.4%)
- Yes	7	(7.7%)	4	(9.8%)	11	(8.3%)
- Missing	3	(3.3%)	4	(9.8%)	7	(5.3%)
Self-reported demographics						
Reported residence						
- Lives in town	46	(50.6%)	19	(46.3%)	65	(49.2%)
- Lives out of town	37	(40.7%)	17	(41.5%)	54	(40.9%)
- Missing	8	(8.8%)	5	(12.2%)	13	(9.9%)
Town size (or nearest)						
- Less than 5,000 people	19	(20.9%)	10	(24.4%)	29	(22.0%)
- 5,000 to 15,000 people	23	(25.3%)	11	(26.8%)	34	(25.8%)
- 15,000 to 50,000 people	26	(28.6%)	8	19.5%)	34	(25.8%)
- More than 50,000 people	15	(16.5%)	7	(17.1%)	22	(16.7%)
- Missing	8	(8.8%)	5	(12.2%)	13	(9.9%)
MMM						
- MM 2 Regional centre	-	-	1	(2.45)	1	(0.8%)
- MM 3 Large rural towns	29	(31.9%)	14	(34.2%)	43	(32.6%)
- MM 4 Medium rural towns	30	(33.0%)	18	(43.9%)	48	(36.4%)
- MM 5 Small rural towns	31	(34.1%)	8	(19.5%)	39	(29.6%)
- MM 6 Remote communities	1	(1.1%)	-	-	1	(0.8%)
Has private health insurance						
- No	41	(45.1%)	15	(36.6%)	56	(42.4%)
- Yes	46	(50.6%)	25	(61.0%)	71	(53.8%)
- Don’t wish to answer/missing	4	(4.4%)	1	(2.4%)	5	(3.8%)
Education						
- Year 12 or less	43	(47.3%)	16	(39.0%)	59	(44.7%)
- Trade or vocation	20	(22.0%)	12	(29.3%)	32	(24.2%)
- University or higher	20	(22.0%)	8	(19.5%)	28	(21.2%)
- Don’t wish to answer/missing	8	(8.8%)	5	(12.2%)	13	(9.9%)
Household income ($AUD)						
- < $700 per week	14	(15.4%)	7	(17.1%)	21	(15.9%)
- $700 - < $1,000 per week	19	(20.9%)	6	(14.6%)	25	(18.9%)
- $1,000 - < $1,500 per week	15	(16.5%)	4	(9.8%)	19	(14.4%)
- $1,500 - < $2,500 per week	13	(14.3%)	6	(14.6%)	19	(14.4%)
- $2,500 or higher per week	10	(11.0%)	10	(24.4%)	20	(15.2%)
- Don’t wish to answer/missing	20	(22.0%)	8	(19.5%)	28	(21.2%)
Living arrangements						
- Self	7	(7.7%)	6	(14.6%)	13	(9.9%)
- Partner	59	(64.8%)	24	(58.5%)	83	(62.9%)
- Children	2	(2.2%)	0	(0.0%)	2	(1.5%)
- Partner & children	11	(12.1%)	4	(9.8%)	15	(11.4%)
- Other	4	(4.4%)	1	(2.4%)	5	(3.8%)
- Don’t wish to answer/missing	8	(8.8%)	6	(14.6%)	14	(10.6%)
Self-reported conditions						
- High cholesterol	49	(53.9%)	29	(70.7%)	78	(59.1%)
- High blood pressure	57	(62.6%)	29	(70.7%)	86	(65.2%)
- Diabetes	19	(20.9%)	10	(24.4%)	29	(22.0%)
- Overweight or obese	37	(40.7%)	21	(51.2%)	58	(43.9%)
- Don’t wish to answer/missing	8	(8.8%)	5	(12.2%)	13	(9.9%)
Self-reported taking medications for:						
- Lowering cholesterol	45	(49.5%)	34	(82.9%)	79	(59.8%)
- Blood pressure control	58	(63.7%)	33	(80.5%)	91	(68.9%)
- Blood glucose control	22	(24.2%)	9	(22.0%)	31	(23.5%)

Key: ^a^Risk of CVD within the next 5 years

**Table 2:** Baseline measures related to outcomes in HealthyRHearts study participants

	Reference range	Intervention (*n* = 91)			Usual Care (*n* = 41)			Total (*n* = 132)		
Lipid values (mmol/L) [61]		n	mean	(SD)	n	mean	(SD)	n	mean	(SD)
- Total cholesterol	< 5.5	91	5.04	(1.11)	41	4.80	(1.18)	132	4.97	(1.13)
- LDL cholesterol	< 3.0	86	2.96	(0.93)	38	2.71	(1.09)	124	2.88	(0.98)
- Triglycerides	< 2.0	91	1.82	(1.07)	40	1.80	(1.13)	131	1.81	(1.08)
Diabetes indicators [61]										
- Blood glucose levels (mmol/L)	3.0 to 6.0 (fasting)	83	6.16	(1.42)	40	6.05	(1.21)	123	6.12	(1.36)
- HbA1c (NGSP unit: % HbA1c)	< 6.0%	83	5.87	(0.82)	36	5.84	(0.71)	119	5.86	(0.78)
Anthropometrics [10]										
- Systolic BP (mmHg)	< 130	88	141.6	(15.7)	37	137.7	(13.6)	125	140.5	(15.1)
- Diastolic BP (mmHg)	< 80	88	82.1	(10.1)	37	80.5	(10.6)	125	81.6	(10.2)
- BMI (kg/m2)	18.5 to 24.9	79	31.7	(7.1)	34	32.3	(5.5)	113	31.9	(6.6)
- Weight (kg)	-	80	98.0	(23.6)	34	93.0	(16.8)	114	94.4	(21.7)
- Waist circumference (cm)										
- Total	-	69	109.5	(15.5)	24	108.2	(13.3)	93	109.1	(14.9)
- Female	> 88 cm	20	104.0	(10.6)	11	107.3	(17.5)	31	105.2	(13.2)
- Male	> 102 cm	49	111.7	(16.7)	13	109.0	(9.2)	62	111.1	(15.4)

### Usual care during the study

During the study, one person in the intervention group passed away from causes unrelated to the study and another underwent bypass surgery. Ninety-eight participants provided information about the number of visits made to their GP for their heart health during their 12-month enrolment (Int: *n* = 69; UC: *n* = 29). Most reported visiting 0–2 times (Int: *n* = 57, 83%; UC: *n* = 25, 86%) with others having 3–6 visits (Int: *n* = 11, 16%; UC: *n* = 4, 14%), and the remainder being unsure. Twenty people additionally reported visiting specialists or allied health providers. Cardiologists were the most visited specialist (Int: *n* = 6; UC *n* = 2), with one UC participant also visiting an endocrinologist. Dietitians and exercise physiologists were the most visited allied health providers. Four intervention participants reported additional dietetic appointments external to the study, and three UC participants reported dietetic visits during their enrolment, with six intervention and one UC participants reporting exercise physiologist referrals. One participant from each group reported podiatry consultations and one UC participant visited with a diabetes educator. For prescriptions of medication classes during the 12 months, our results suggest that both groups increased prescriptions, with a 60% posterior probability (Bayesian probability that accounts for new information) that the UC group increased medications by 15%, compared to the intervention increase of 9.5% (see Supplementary material three).

### Primary outcome– total cholesterol

The mean change in total cholesterol from baseline to 12-month follow-up was −0.28 (0.79) for the UC group and −0.26 (0.77) for the intervention group. The adjusted difference in mean change in total cholesterol between the groups (intervention minus UC) was estimated at 0.14 (95%CI: −0.14, 0.43). The posterior probability that this difference was negative was 0.17, indicating a greater reduction in total cholesterol in the intervention group compared to the UC (see Table 3). Total cholesterol was 0.58 mmol/L higher at baseline (95%CI: 0.22, 0.94) in those who self-reported having high cholesterol as a condition. For each class of cholesterol-lowering medication taken, total cholesterol was lower by −0.98 mmol/L (95%CI: −1.22, −0.75), with total cholesterol at baseline lower by 0.05 mmol/L for each year of age (95%CI: −0.08, −0.01).

### Secondary outcomes

For LDL cholesterol, the mean change from baseline to 12 months was −0.22 (0.80) for the UC group and −0.19 (0.74) for the intervention group. The estimated adjusted difference in mean change between groups (intervention minus UC) was 0.11 (95%CI: −0.14, 0.36). The posterior probability that this difference was negative was 0.80, indicating a greater reduction in LDL cholesterol in the intervention group compared to the control. For triglycerides, the mean change from baseline to 12 months was −0.10 (0.72) in the UC group and −0.28 (0.71) in the intervention group. The estimated difference in mean change between groups (intervention minus UC) was −0.20 (95% CI: −0.46 to 0.05), with a 94% probability that this effect was negative, indicating a likely greater decrease in triglycerides in the intervention group. Both LDL cholesterol and triglycerides were lower with older age (see Table 3).

There was very little evidence of a difference in mean change from baseline to 12 months in either systolic or diastolic blood pressure (see Table 4), with posterior probabilities of a greater decrease in the intervention group compared to UC of 0.62 for systolic and 0.18 for diastolic pressure.

Mean fasting blood glucose concentrations decreased from baseline to 12 months in the intervention group by −0.19 mmol/L (0.81) and increased in the UC group by 0.05 mmol/L (1.11), with a 95% probability that this change was greater in the intervention compared to UC (adjusted difference in change from baseline: −0.32; 95% CI: −0.70 to 0.06). Participants with diabetes had higher baseline levels of blood glucose by 1.16 mmol/L (95% CI: 0.73 to 1.61) and HbA1c by 0.82% (NGSP)(95% CI: 0.55 to 1.10). HbA1c levels improved in the intervention group compared to baseline, with a mean change of −0.11% (0.39) at 12 months and increased in UC, with a mean change 0.41 (0.38). There was a 98% probability that the difference was greater for intervention compared to UC (adjusted difference in change from baseline −0.16%; 95% CI: −0.32, −0.01).

Mean weight change between baseline and 12-months for the intervention group was −2.04 kg (3.81) and 0.03 kg (2.43) for the UC group. There was a 99% probability that the weight loss was greater for the intervention group at 12 months (adjusted difference in change from baseline −2.46 kg, 95%CI: −4.54, −0.41) (see Table 4). Additionally, for each increase in percent of energy from nutrient-dense, core foods, a 60 g decrease in body weight was found (−0.06, 95%CI: −0.12, −0.00). A similar result was found for waist circumference, with a 0.12 cm decrease for each percent of energy from core foods (−0.12, 95%CI: −0.23, −0.01).

**Table 3:** Results from the primary outcome of total cholesterol, with estimates derived from bayesian mixed models, incorporating MCMC sampling with four chains of 10,000 iterations and random intercepts for the individual and the primary care practice from which they were recruited

		Total cholesterol (mmol/L)		LDL cholesterol (mmol/L)		Triglycerides (mmol/L)		Systolic BP (mm/Hg)		Diastolic BP (mm/Hg)	
Variable	Level or statistic	Est.	95% CI	Est.	95% CI	Est.	95% CI	Est.	95% CI	Est.	95% CI
Intercept		8.22	6.19, 10.22	5.66	4.10, 7.19	3.98	2.07, 5.83	146.69	123.03, 170.28	94.41	77.39, 111.07
Age	Years	−0.05	− 0.08, − 0.01	− 0.04	− 0.06, − 0.01	− 0.04	− 0.07, − 0.01	−0.31	−0.69, 0.07	− 0.34	− 0.61, − 0.07
Gender	Female	(ref)		(ref)		(ref)		(ref)		(ref)	
	Male	−0.21	−0.56, 0.15	−0.11	−0.39, 0.18	0.06	−0.29, 0.41	6.87	2.57, 11.21	2.87	−0.19, 5.93
Self-reported condition^a^	Yes	0.58	0.22, 0.94	0.37	0.10, 0.65	-	-	11.91	6.90, 17.00	6.87	3.29, 10.37
Medication^b^ for condition management	Number of medication classes taken	− 0.98	− 1.22, − 0.75	− 1.01	− 1.20, − 0.83	−0.36	−0.89, 0.18	−1.72	−4.10, 0.59	0.73	−0.90, 2.33
Time^1^	Baseline (time 0)	(ref)		(ref)		(ref)		(ref)		(ref)	
	3 months	− 0.29	− 0.55, − 0.02	−0.16	−0.38, 0.07	−0.02	−0.25, 0.21	-	-	-	-
	6 months	−0.10	−0.35, 0.15	−0.05	−0.27, 0.17	−0.11	−0.33, 0.11	-	-	-	-
	12 months	−0.21	−0.45, 0.03	−0.12	−0.33, 0.09	−0.01	−0.23, 0.20	−3.65	−10.01, 2.81	0.71	−3.62, 5.09
Group^2^	Usual care	(ref)		(ref)		(ref)		(ref)		(ref)	
	Intervention	0.06	−0.50, 0.61	0.05	−0.40, 0.51	0.11	−0.32, 0.54	4.53	−1.03, 10.21	2.21	−1.90, 6.20
Group # Time^3^	Baseline # Usual care group	(ref)		(ref)		(ref)		(ref)		(ref)	
	3 m # Int. group	0.23	−0.08, 0.54	0.14	−0.12, 0.41	−0.23	−0.49, 0.05	-	-	-	-
	6 m # Int. group	0.12	−0.18, 0.41	0.14	−0.12, 0.40	−0.13	−0.39, 0.14	-	-	-	-
	12 m # Int. group	0.14	−0.14, 0.43	0.11	−0.14, 0.36	−0.20	−0.46, 0.05	1.23	−6.39, 8.77	−2.39	−7.70, 2.69
Post. Prob^4^	12 m # Int. group	0.83		0.80		0.06		0.62		0.18	
Evid. Ratio^5^	12 m # Int. group > 0	4.97		4.08		0.06		1.66		0.22	

Also included are related lipid secondary outcomes of LDL cholesterol, serum triglycerides and blood pressure

Key: *Int* Intervention, *m* Months

^a^Self-reported conditions were high cholesterol for total and LDL cholesterol, high blood pressure for systolic and diastolic blood pressure

^b^See [medication classes– supplementary material for specific classes included]

1. Parameter estimates are the mean change from baseline for the control group at each follow up time point

2. Parameter estimate compares the mean outcome at baseline between intervention and control

3. The key parameter of interest, representing the difference in mean change from baseline between intervention and control

4. The probability the parameter is greater than zero for positive estimates, or less than zero for negative estimates (the probability of direction (pd))

5. Evidence ratio (pd/1-pd)

**Table 4:** Results from the secondary outcomes of glucose control, weight and waist circumference, with estimates derived from bayesian mixed models, incorporating MCMC sampling with four chains of 10,000 iterations and random intercepts for the individual and the primary care practice from which they were recruited from

		Blood glucose (fasting) (mmol/L)			Hba1c (NGSP %)		Weight (kg)			Waist circumference (cm)	
Variable	Level or statistic	Est.	95% CI		Est.	95% CI	Est.		95% CI	Est.	95% CI
Intercept		4.97	3.18, 6.80		6.21	5.13, 7.32	84.13		40.66, 128.10	119.77	85.81, 154.63
Age	Years	0.01	−0.02, 0.03		−0.01	−0.03, 0.01	−0.19		−0.87, 0.47	−0.20	−0.74, 0.33
Gender	Female	(ref)			(ref)		(ref)			(ref)	
	Male	0.30	−0.02, 0.63		−0.03	−0.24, 0.17	19.66		11.82, 27.52	7.45	1.67, 13.28
Self-reported condition^a^	Yes	1.16	0.73, 1.61		0.82	0.55, 1.10	21.69		14.65, 28.76	12.25	6.91, 17.53
Medication^b^ for condition management	Number of medication classes taken	0.40	0.21, 0.59		0.15	0.04, 0.27	-		-	-	-
Education	Year 12 or less						(ref)				
	Trade or vocation	-	-		-	-	5.05		−3.24, 13.40	-	-
	University or higher	-	-		-	-	0.45		−8.07, 8.86	-	
Diet	% energy core foods	-	-		-	-	− 0.06		− 0.12, − 0.00	− 0.12	− 0.23, − 0.01
Physical activity	(min/week)	-	-		-	-	-		-	−0.00	−0.00, 0.00
Time^1^	Baseline	(ref)			(ref)		(ref)			(ref)	
	3 m	−0.13	−0.46, 0.21		−0.03	−0.17, 0.11	−0.73		−2.90, 1.43	− 3.96	− 7.57,− 0.33
	6 m	−0.18	−0.52, 0.15		−0.02	−0.15, 0.11	−1.14		−3.21, 0.94	−2.92	−6.67, 0.90
	12 m	0.05	−0.27, 0.36		0.04	−0.10, 0.17	0.61		−1.24, 2.51	−2.32	−5.91, 1.36
Group^2^	Usual care	(ref)			(ref)		(ref)			(ref)	
	Intervention	0.21	−0.37, 0.84		0.13	−0.13, 0.40	0.53		−7.83, 8.72	−1.09	−7.76, 5.35
Group # Time^3^	Baseline # Usual care.	(ref)			(ref)						
	3 m # Int.	0.03	−0.37, 0.43		−0.07	−0.24, 0.09	0.38		−2.06, 2.82	1.84	−2.41, 6.01
	6 m # Int.	−0.02	−0.42, 0.37		−0.08	−0.24, 0.08	−0.62		−2.95, 1.67	−0.02	−4.37, 4.29
	12 m # Int.	−0.32	−0.70, 0.06		13096	− 0.32,− 0.01	− 2.46		− 4.54,− 0.41	−0.88	−5.07, 3.34
Post. Prob^4^	12 m # Int. group	0.05			0.02		0.01			0.34	
Evid. Ratio^5^	12 m # Int. group > 0	0.05			0.02		0.01			0.51	

*Key*: -: not used in model, *BP* Blood pressure, *Evid. Ratio* Evidence ratio, *Est* Estimate, *Int* Intervention group, *m* months, *Post. Prob* Posterior probability, *UC* Usual care group

^a^Self-reported conditions were high blood pressure for systolic and diastolic blood pressure, diabetes (any type) diagnosis glucose-related outcomes and overweight/obesity for weight and waist circumference

^b^See [medication classes– supplementary material for specific classes included]

1. Parameter estimates are the mean change from baseline for the control group at each follow up time point

2. Parameter estimate compares the mean outcome at baseline between intervention and control

3. The key parameter of interest, representing the difference in mean change from baseline between intervention and control

4. The probability the parameter is greater than zero for positive estimates, or less than zero for negative estimates (the probability of direction (pd))

5. Evidence ratio (pd/1-pd)

## Discussion

This is the first rural Australian telehealth MNT intervention delivered to evaluate individual factors contributing to CVD risk in a pragmatic randomised controlled trial. The pragmatic inclusion of participants at moderate to high risk of CVD reflected real-world populations, which incorporated a range of medicated and unmedicated health conditions and co-morbidities at varying levels. While no significant improvements were found in total cholesterol between the intervention and control group, the intervention group had significant improvements in HbA1c and body weight at the 12-month follow-up, compared to usual care. Like many other interventions that commenced during the COVID-19 pandemic, study findings were impacted by a substantially reduced sample size. Despite this challenge, the study has produced considerable evidence for clinically relevant dietary and health changes through the delivery of two hours of dietitian intervention via telehealth over a 12-month period.

The effectiveness of MNT interventions on change in cardiometabolic outcomes including anthropometrics, plasma lipids and HbA1c responses varies [31, 32, 36, 62, 63]. The lack of difference between groups in total cholesterol at 12-months may be explained by a range of factors, including effective management following a heart health check by GPs as a part of usual care and/or a high compliance with medication by those who identified high cholesterol as a health condition. Prior studies have shown that cholesterol lowering medications have a much greater effect on reducing cholesterol levels, whereas to achieve a similar reduction, multiple lifestyle interventions and a ≥ 5 kg weight reduction may be required to achieve the same effect as statin monotherapy [64]. However, it is noteworthy that improvements in dietary intake and quality may result in several other health- and CVD-related benefits [65, 66], whereas statin monotherapy only targets cholesterol levels and has shown to impede diet quality [67]. Additionally, total cholesterol levels in the usual care group were maintained throughout the study. Therefore, any reductions in total cholesterol because of improved dietary intake may have been too small to achieve significant results.

Lifestyle interventions are recommended in guidelines as first line therapy with or without pharmacotherapy for lowering cholesterol [34], although in some cases pharmacotherapy may be initiated in place of lifestyle advice due to its greater impact on cholesterol levels. In the United States, population data from 1999 to 2010 has shown a 14.4% increase of fat intake in statin users [68], perhaps indicating that statin prescription and lifestyle advice need to be concurrent. If left unattended, total cholesterol is anticipated to increase each year with age [69]; however, this did not happen in either group, suggesting effectiveness of a heart health assessment by a GP. A previous Australian study found that assessment of cardiovascular risk with a health professional, followed by a discussion of risk factors, which could include dietary advice, can reduce absolute risk over a 12-month period [70].

The evidence regarding effectiveness of MNT on lipid outcomes is less consistent, with two meta-analyses showing no significant differences [31, 36]. One meta-analysis specific to telehealth showed a small, but significant decrease in the intervention arm receiving dietary advice on reduction in total cholesterol levels (MD: −0.08mmol/L, 95% CI: −0.16, −0.00), but not on other lipid outcomes [71]. This is dissimilar to a meta-analysis on MNT, which found that compared to usual care or no intervention, MNT significantly reduced total cholesterol, LDL, triglycerides, and systolic blood pressure [72]. The interventions (*n* = 7) included in the latter meta-analysis were between 6-weeks and 12-months and provided between two and 12 (mostly > 5) in-person contacts (30–120 min in duration) [72]. The current study prescribed five, brief MNT telehealth consults with an APD over the 12-month period (two hours of time in total), as this reflects what patients could potentially currently access under Australia’s Medicare funded chronic disease management plan scheme ^(73)^, and was conducted using telehealth. It must be noted that the effectiveness of the timing of the scheduled consultations and the time limits applied were not investigated as part of this study, and that different allocations, including extended MNT intervention periods, may be more beneficial. Overall, the current MNT intervention resulted in small changes in some cardiometabolic outcomes. Further research could consider the benefits of overall small changes in many cardiometabolic health outcomes for the prevention of CVD in the long term.

For the secondary outcomes, there was a significant improvement in HbA1c in the intervention compared to control group at the 12-month time-point and fasting blood glucose approached statistical significance. The mean HbA1c and blood glucose levels at baseline were in the pre-diabetic range in both the intervention and control groups, with approximately 22% of participants reporting diabetes as a condition and 24% taking blood glucose control medications. The prevalence of T2D in rural areas is 1.3 times greater compared to individuals living in major cities [6], and access to services is scarcer. Therefore, MNT may be a promising intervention to more efficiently manage T2D in rural areas. It is possible that the indicated improvements in glycaemic control were secondary to weight loss, rather than due to medication, given the significant reduction in body weight in the intervention group at 12 months compared to control. Meta-analyses have shown that dietary interventions result in small but significant reductions in body weight (WMD − 0.8 kg to − 1.71 kg [36, 71, 73]), highlighting the importance of the current study with a greater mean reduction in weight of − 2.5 kg in intervention group participants. Further, current obesity management guidelines suggest a weight loss of 3–5% is expected from behavioural interventions [74], consistent with the ~ 2% mean weight reduction observed in the current study, where weight loss was not a nominated goal for the whole cohort. While no meta-analyses have been conducted assessing the effectiveness of MNT on HbA1c outcomes outside of people with diabetes, results consistently show that MNT can effectively reduce HbA1c levels [32, 62]. Prior meta-analysis in populations with similar baseline Hba1c values has shown that MNT can reduce HbA1c levels by 0.30% over three to 24 months [75], with the current study reporting an estimated reduction of 0.11% over 12 months. This reduction is clinically important as estimations have indicated that a 0.1% reduction in HbA1c in the general population could prevent 12% of excess mortality associated with glycated haemoglobin [76]. Even though HbA1C reduction was not the goal of the MNT intervention, the significant reduction in HbA1c levels post MNT intervention was to be expected and adds to the current evidence base supporting the benefits of MNT on glycaemic outcome and its recommendation as part of chronic disease risk reduction broadly.

The COVID-19 pandemic significantly impacted the HealthyRHearts study protocol, particularly in terms of sample size, recruitment and operational strategies. The primary care practices and GPs faced unprecedented challenges due to the pandemic [56] and it was possibly the key reason why planned sample size was not reached. Many primary care practices were overwhelmed with demand for vaccinations and staffing challenges during the pandemic, with the trial placing a further burden on their resources and limited their capacity to engage in research activities. This consequently placed a strain on the recruitment processes and the trial had to be extended to adapt to the new realities imposed by the pandemic. While the pandemic made many aspects of the study challenging and required accommodations to public health orders and conditions at the time, the MNT interventions were able to be conducted, with the telehealth delivery being an asset.

Rural populations are under-represented in health research, despite experiencing health inequities and disproportionally greater burden of chronic disease [77]. There is also a lack of research funding directed to improve nutrition. Therefore, further funding opportunities and research are needed to improve nutrition related health within rural areas of Australia [78, 79]. Additionally, further effort needs to be made to understand the effect of this intervention on overall CVD risk, glycaemic control and in-depth nutritional and dietetic outcomes.

### Study strengths and limitations

A major strength of the current study is that it is the first study to evaluate MNT for reduction of CVD risk factors delivered in a rural setting using telehealth. Telehealth is a promising format for delivery of MNT and was not impeded by the COVID-19 pandemic. Another strength is that the HealthyRHearts study used a scalable real-world approach, which can be integrated into the current primary care system and could be expanded through dedicated Medicare funded MNT care plans for chronic disease. Compared with other studies using MNT, the current study offered two hours of MNT, delivered through five sessions during a 6-month period to participants, which reflects what may be available under the Australian Medicare system for chronic disease management [80].

One key limitation was the inability to reach the planned sample size for reasons discussed above, and the resulting effect on clusters within the study. The results were underpowered and should therefore be interpreted with caution. While it is difficult to quantify the impact of COVID-19 on this study, we used an amended statistics plan that has been approved for studies affected by COVID-19. However, an increased sample size would have allowed for adjusting in a greater number of demographics, such as income or private health insurance. Also, we did not collect demographics related to cultural diversity and are unable to compare our study sample to the wider population.

### Recommendations for primary care practices

- Referral to an APD for MNT may result in many small, but clinically relevant changes in multiple comorbidities and health outcomes for individuals at an elevated risk of CVD, including reductions in HbA1c and weight.
- While a significant between group reduction in lipid levels was not achieved, there is enough evidence to show that lifestyle interventions and MNT interventions can significantly support usual care offered by GPs. Further, clinical practice guidelines support lifestyle interventions, including changes in diet, to be used as first line therapy for the management of CVD risk factors. Therefore, referral to an APD prior to or in conjunction with usual care should be encouraged in individuals identified to be at an elevated CVD risk.
- Heart health checks and discussion of risk factors are a useful strategy to reduce 12-month CVD risk and can result in improved health outcomes, particularly related to glycaemia and body weight. It is recommended that heart health checks are undertaken regularly in high-risk populations.

## Conclusion

This was the first telehealth MNT intervention conducted in rural Australia in participants at an elevated risk of CVD. Significant improvements in the intervention group were identified for HbA1c, weight and waist circumference but not for lipids, in comparison to usual care. Further research on the application of MNT in rural areas is required, however currently these results are encouraging to support the promotion of MNT as a synergistic adjunct therapy to usual care provided by GPs for individuals at increased cardiovascular risk.

## Supplementary Information

Supplementary Material 1.

Supplementary Material 2.

Supplementary Material 3.

Supplementary Material 4

## Data Availability

Available upon request at the discretion of the corresponding author, Laureate Professor Clare Collins. Email: Clare.Collins@newcastle.edu.au.
