# Supplementary figures and images for: The effectiveness of medical nutrition therapy for people at moderate to high risk of cardiovascular disease in an Australian rural primary care setting: 12-month results from a pragmatic cluster randomised controlled trial
Source: BMC Health Serv Res. 2025 Jul 16;25:956. doi: 10.1186/s12913-025-13096-8 (PMC12269237; doi:10.1186/s12913-025-13096-8)

**Supplementary material 1: Example dietary feedback form**

**
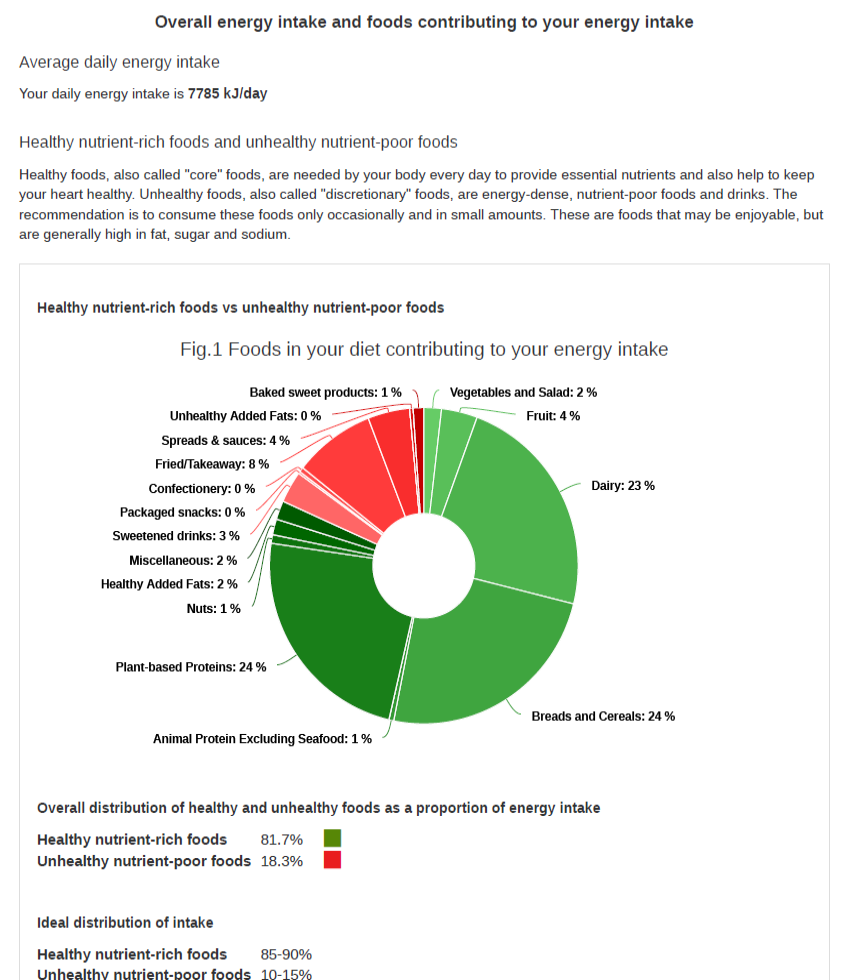
**

Supplement: Supplementary file 1 — Supplementary Material 1. [file 12913_2025_13096_MOESM1_ESM.docx]
