# Supplementary material for: The effectiveness of medical nutrition therapy for people at moderate to high risk of cardiovascular disease in an Australian rural primary care setting: 12-month results from a pragmatic cluster randomised controlled trial
Source: BMC Health Serv Res. 2025 Jul 16;25:956. doi: 10.1186/s12913-025-13096-8 (PMC12269237; doi:10.1186/s12913-025-13096-8)
Supplement: Supplementary file 2 — Supplementary Material 2. [file 12913_2025_13096_MOESM2_ESM.docx]

**Supplementary material three: Changes in medication classes, controlled by age, gender and number of self-reported health conditions**

|  | | **Medication classes** | |
| --- | --- | --- | --- |
| **Variable** | **Level or statistic** | **Incident rate ratio** | **95% CI** |
| Intercept |  | 0.74 | 0.12, 4.56 |
| Age | Years | 1.01 | 0.98, 1.03 |
| Gender | Female | (ref) | |
|  | Male | 1.05 | 0.76, 1.46 |
| Health conditions | Number of self-reported health conditions | 1.35 | 1.18, 1.55 |
| Time^1^ | Baseline (time 0) | (ref) | |
|  | 3 months | 1.08 | 0.81, 1.46 |
|  | 6 months | 1.06 | 0.78, 1.44 |
|  | 12 months | 1.14 | 0.85, 1.51 |
| Group^2^ | Usual care | (ref) | |
|  | Intervention | 0.90 | 0.56,1.48 |
| Group # Time^3^ | Baseline # Usual care group | (ref) | |
|  | 3m # Int. group | 0.93 | 0.65, 1.35 |
|  | 6m # Int. group | 0.96 | 0.66, 1.40 |
|  | 12m # Int. group | 0.95 | 0.66, 1.35 |
| Evidence ratio | 12 months # Intervention group >0 | 1.60 | |
| Posterior probability | 12 months # Intervention group | 0.62 | |
| 1. Parameter estimates are the mean change from baseline for the control group at each follow up time point 2. Parameter estimate compares the mean outcome at baseline between intervention and control 3. The key parameter of interest, representing the difference in mean change from baseline between intervention and control | | | |
