# Supplementary material for: The effectiveness of medical nutrition therapy for people at moderate to high risk of cardiovascular disease in an Australian rural primary care setting: 12-month results from a pragmatic cluster randomised controlled trial
Source: BMC Health Serv Res. 2025 Jul 16;25:956. doi: 10.1186/s12913-025-13096-8 (PMC12269237; doi:10.1186/s12913-025-13096-8)
Supplement: Supplementary file 3 — Supplementary Material 3. [file 12913_2025_13096_MOESM3_ESM.docx]

**Supplementary material two:** A schedule of primary and secondary outcome data collected at the study time points of baseline, 3-, 6- and 12-months, and their assessment details.

| **Data collection and dietetic consultation schedule** | | | | | | |
| --- | --- | --- | --- | --- | --- | --- |
|  | **Baseline** | **Review 1**  **(+ 2 weeks)** | **Review 2**  **(+ 4 weeks)** | **3 months** | **6 months** | **12 months** |
| **Pathology (fasting)** | | | | | | |
| Total cholesterol, LDL and HDL cholesterol, triglycerides, glucose & Hba1c | 🗹 |  |  | 🗹 | 🗹 | 🗹 |
| **Medications** | | | | | | |
| Reported to recruiting officer | 🗹 |  |  |  |  | 🗹 |
| Self-report changes via survey |  |  |  | 🗹 | 🗹 | 🗹 |
| **Anthropometry** | | | | | | |
| ***Measured by GP / primary care practice staff*** |  |  |  |  |  |  |
| Blood pressure | 🗹 |  |  |  |  | 🗹 |
| Weight & waist circumference | 🗹 |  |  |  |  | 🗹 |
| Height | 🗹 |  |  |  |  |  |
| ***Self-report by participant*** |  |  |  |  |  |  |
| Weight & waist circumference | 🗹 |  |  | 🗹 | 🗹 | 🗹 |
| Height | 🗹 |  |  |  |  |  |
| **Participant surveys** | | | | | | |
| Demographics | 🗹 |  |  |  |  |  |
| **Dietetic consultation schedule** | | | | | | |
| Personalised Nutrition Questionnaire | 🗹 |  |  |  |  |  |
| Dietary surveys (AES-Heart) | 🗹 |  |  | 🗹 | 🗹 | 🗹 |
| Length | 30 minutes | 20 minutes | 20 minutes | 20 minutes | 30 minutes | n/a |
| Content | - Review AES-Heart analysis & reports - Make SMART goals - Distribute resources | - Review SMART goals & adapt if needed - Discuss barriers & enablers - Distribute resources | - Review SMART goals & adapt if needed - Discuss barriers & enablers - Distribute resources | - Review AES-Heart analysis - Review SMART goals & adapt if needed - Discuss barriers & enablers - Distribute resources | - Review AES-Heart analysis - Review SMART goals & adapt if needed |  |

*Pathology*

The primary outcome of total cholesterol, and secondary outcomes of cholesterol fractions, triglycerides, glucose and HbA1c were measured using fasting blood samples, which were collected and analysed by an accredited pathology lab. The LDL value was calculated indirectly using the Friedewald formula, not measured directly. Three pathology services were required to cover the study geographical of rural participants. All collection sites were asked to use citrate fluoride tubes to prevent glycolysis, and account for distance between collection point and analysis at the nearest laboratory.

*Anthropometry*

GPs/primary care practice staff were asked to collect blood pressure, weight and waist circumference measures at the initial assessment and again at the subsequent annual heart health check. Height was only requested at the initial visit.

Participants were asked to report an initial height measure at baseline, and weight and waist circumference measures at baseline, 3-, 6- and 12-months. They were provided with written and online video instructions on how to correctly take measurements and were asked to take all measures twice at each time point.

To maximize included data, weight and waist circumference measures were used from GP and participant sources. However, only one source was used per participant, to ensure consistency of measure. For example, three or four self-reported values were preferred over two GP measures, and two GP measures were preferred over two self-report values, unless a data entry error was suspected.

*Demographic data*

Participants were asked to self-report demographics for where they lived, their highest level of education, living arrangements, household income, and to identify whether they had ever been told by a health professional that they had any of the 11 listed health conditions related to heart health or that are commonly reported among Australian adults ^(76)^. All questions allowed for a “do not wish to answer” response. Education, living arrangements and income categories were collapsed for reporting purposes, and to protect anonymity of low response answers. Participants reporting they received a pension as their income were nominated a value based on their response to their living arrangements (i.e. a single person or couple) and according to Australian age pensions ^(77)^.

*Access to healthcare services during the follow-up period*

Towards completion of participant’s 12-month follow-up period, a recruitment officer attempted to contact all participants who had not withdrawn from the study or had been lost to contact. Where possible, the officer confirmed current medications or any changes that may have occurred throughout the year, the estimated number of visits to their GP about heart health and whether they had seen any other health professionals in relation to heart health.

**Medications**

Participant medications were recorded at baseline, 3-, 6- and 12-months. GPs were asked to provide a list of current medications affecting heart health at both the initial and annual visit, in addition to recruiting officers asking participants which medications they were taking. The Anatomical Therapeutic Chemical (ATC) classification was used to code participant medicines for analysis ^(78)^. Only medicine classes indicated for the study primary or secondary outcomes were included (that is, medicines indicated for the treatment of hyperlipidaemia, hypercholesterolemia, hypertriglyceridemia, hypertension, diabetes, and weight reduction).

**Medication coding**

Within the included medicine classes, medicines that were being taken for other indications were included for analysis (for example, a beta-blocker taken for migraine). Medicines that had possible adverse reactions affecting a primary or secondary outcome were excluded from analysis (see Supplementary Material for complete list of included and excluded medicine classes).

For each medicine, the prescribed dose was calculated as a proportion of the defined daily dose (DDD) ^(78)^. The DDD is the assumed average maintenance dose per day for a medicine, based on its main indication in adults. For variable insulin doses, no dose was provided. For combination medicines, individual medicine doses were used to calculate the DDD proportion to differentiate between various strengths of combination products.

Dosage discrepancies between the participant report and clinician report were reviewed and resolved on a case-by-case basis. Clarification was requested where possible; when clarification was not possible or did not resolve the discrepancy, a team discussion was held, and a decision made based on the information available. If a clinician reported prescribing a medicine but the patient reported not taking the medicine, the medicine was not included in the analysis.

| **STUDY OUTCOME** | **MEDICINE CLASS^1^ AFFECTING OUTCOME^2^** | **EXAMPLE** | **CODE** |
| --- | --- | --- | --- |
| ***PRIMARY OUTCOMES*** | | | |
| Total serum cholesterol | HMG CoA reductase inhibitors | Atorvastatin | C10AA |
|  | Fibrates | Fenofibrate | C10AB |
|  | Other lipid modifying agents | Ezetimibe | C10AX |
|  | Combinations of various lipid modifying agents | Rosuvastatin and ezetimibe | C10BA |
| Serum low density lipoprotein (LDL) cholesterol | Fibrates | Fenofibrate | C10AB |
| Serum triglycerides | Fibrates | Fenofibrate | C10AB |
| ***SECONDARY OUTCOMES*** | | | |
| Systolic and diastolic blood pressure | Alpha-adrenoreceptor antagonists | Prazosin | C02CA |
|  | Pyrimidine derivatives | Minoxidil | C02DC |
|  | Thiazides, plain | Hydrochlorothiazide | C03AA |
|  | Sulfonamides, plain | Chlortalidone | C03BA |
|  | Sulfonamides, plain | Furosemide | C03CA |
|  | Aldosterone antagonists | Spironolactone | C03DA |
|  | Beta blocking agents, non-selective | Sotalol^3^ | C07AA |
|  | Beta blocking agents, selective | Metoprolol, bisoprolol^4^ | C07AB |
|  | Alpha and beta blocking agents | Carvedilol^5^ | C07AG |
|  | Dihydropyridine derivatives | Amlodipine | C08CA |
|  | Phenylalkylamine derivatives | Verapamil | C08DA |
|  | ACE^6^ inhibitors, plain | Perindopril | C09AA |
|  | ACE inhibitors and diuretics | Perindopril and indapamide | C09BA |
|  | ACE inhibitors and calcium channel blockers | Perindopril and amlodipine | C09BB |
|  | Angiotensin II receptor blockers (ARBs), plain | Candesartan | C09CA |
|  | Angiotensin II receptor blockers (ARBs) and diuretics | Irbesartan and hydrochlorothiazide | C09DA |
|  | Angiotensin II receptor blockers (ARBs) and calcium channel blockers | Telmisartan and amlodipine | C09DB |
|  | Angiotensin II receptor blockers (ARBs), other combinations | Valsartan and amlodipine and hydrochlorothiazide | C09DX |
|  | Angiotensin II receptor blockers (ARBs) and calcium channel blockers | Telmisartan and amlodipine | C09DB |
| ***OTHER*** | | | |
| Blood glucose level | Insulins and analogues for injection, fast-acting^7^ | Insulin aspart | A10AB |
|  | Insulins and analogues for injection, intermediate- or long-acting combined with fast-acting^7^ | Insulin degludec and insulin aspart | A10AD |
|  | Insulins and analogues for injection, long-acting^7^ | Insulin glargine | A10AE |
|  | [Biguanides](https://atcddd.fhi.no/atc_ddd_index/?code=A10BA&showdescription=no)^7^ | Metformin | A10BA |
|  | Sulfonylureas^7^ | Gliclazide | A10BB |
|  | Combinations of oral blood glucose lowering drugs^7^ | Metformin and empagliflozin | A10BD |
|  | Dipeptidyl peptidase 4 (DPP-4) inhibitors^7^ | Sitagliptin | A10BH |
|  | [Glucagon-like peptide-1 (GLP-1) analogues](https://atcddd.fhi.no/atc_ddd_index/?code=A10BJ&showdescription=no)^7^ | Semaglutide, liraglutide^8^ | A10BJ |
|  | Sodium-glucose co-transporter 2 (SGLT2) inhibitors^7^ | Empagliflozin | A10BK |
| 1. *According to the Anatomical Therapeutic Chemical (ATC) classification, available at* [*https://atcddd.fhi.no/*](https://atcddd.fhi.no/) 2. *Only indications (i.e. uses) registered with the Australian Government Therapeutic Goods Administration (TGA) considered* 3. *Sotalol is indicated for arrhythmias but is classified with other non-selective beta blocking agents; indicated for hypertension at class level.* 4. *Bisoprolol is indicated for heart failure but is classified with other selective beta blocking agents; indicated for hypertension at class level.* 5. *Carvedilol is indicated in heart failure but is classified with other alpha and beta blocking agents; indicated for hypertension at class level.* 6. *ACE: Angiotensin-Converting Enzyme* 7. *Registered indication is treatment of diabetes mellitus* 8. *Liraglutide is indicated for weight reduction but is classified with other GLP-1 analogues; indicated for glycaemic control at class level. NB At the time of data collection, liraglutide was the only GLP-1 analogue registered in Australia for use in weight management* | | | |
